# Hypoxia-inducible factor (*HIF1α*) gene expression in human shock states

**DOI:** 10.1186/cc11414

**Published:** 2012-07-10

**Authors:** Julien Textoris, Nathalie Beaufils, Gabrielle Quintana, Amin Ben Lassoued, Laurent Zieleskiewicz, Sandrine Wiramus, Valéry Blasco, Nathalie Lesavre, Claude Martin, Jean Gabert, Marc Leone

**Affiliations:** 1 Service d'anesthésie et de réanimation, Hôpital Nord, Assistance Publique-Hôpitaux de Marseille, Chemin des bourrely, 13915, Marseille, France; 2 URMITE, CNRS U7278, INSERM U1095, Faculté de médecine Timone, Aix-Marseille Univ, Boulevard Jean Moulin, 13385, Marseille, France; 3 Laboratoire de Biochimie et Biologie Moléculaire, Hôpital Nord, Assistance Publique-Hôpitaux de Marseille, Chemin des bourrely, 13915, Marseille, France; 4 Centre d'Investigation Clinique, Hôpital Nord, Assistance Publique-Hôpitaux de Marseille, Chemin des bourrely, 13915, Marseille, France

## Abstract

**Introduction:**

Hypoxia-inducible factor-1 (HIF1) controls the expression of genes involved in the cellular response to hypoxia. No information is available on its expression in critically ill patients. Thus, we designed the first clinical study in order to evaluate the role of *HIF1α *as a prognosis marker in patients suffering from shock.

**Methods:**

Fifty consecutive adult patients with shock and 11 healthy volunteers were prospectively enrolled in the study. RNA was extracted from whole blood samples and expression of *HIF1α *was assessed over the first four hours of shock. The primary objective was to assess *HIF1α *as a prognostic marker in shock. Secondary objectives were to evaluate the role of *HIF1α *as a diagnostic and follow-up marker. Patient survival was evaluated at day 28.

**Results:**

The causes of shock were sepsis (78%), hemorrhage (18%), and cardiac dysfunction (4%). *HIF1α *expression was significantly higher in the shock patients than in the healthy volunteers (121 (range: 72-168) *versus *48 (range: 38-54) normalized copies, *P *<0.01), whatever the measured isoforms. It was similar in non-survivors and survivors (108 (range 84-183) *versus *121(range 72-185) normalized copies, *P *= 0.92), and did not significantly change within the study period.

**Conclusions:**

The present study is the first to demonstrate an increased expression of *HIF1α *in patients with shock. Further studies are needed to clarify the potential association with outcome. Our findings reinforce the value of monitoring plasma lactate levels to guide the treatment of shock.

## Introduction

Shock states are defined by an acute circulatory failure leading to prolonged and intense tissue hypoxia that may lead to death. Tissue hypoxia is accompanied by a decreased production of ATP in the mitochondria. Hypoxia-inducible factor-1 (HIF1) is a heterodimer made of two sub-units (α and β) [1,2]. The gene coding for HIF1α is on chromosome 14 (14q21-q24) [3]. HIF1α protein concentration is correlated to cellular oxygen concentration [4]. In hypoxemic conditions, HIF1α is not degraded and accumulates in the cellular nucleus [5]. The effects of HIF1α are stimulation of erythropoiesis, glycolysis, angiogenesis, and vasodilation [1]. In normoxic conditions, HIF1α and its messenger RNA (mRNA) have a very short half-life of five minutes [6,7]. This suggests that HIF1α is an immediate surrogate marker of cellular oxygenation.

In human shock states, plasma lactate is routinely used as a marker of tissue hypoxia. This marker has been validated for the detection of shock states as well as the prediction of patient outcomes [8,9]. However, plasma lactate concentrations are influenced by both the production and clearance of lactate. This can be a limitation for the interpretation of plasma lactate concentrations at the bedside. The main objective of the present study was to evaluate the potential prognostic role of *HIF1α *in ICU patients with shock states. Secondary objectives were to evaluate the role of *HIF1α *as a detection marker and its correlation with plasma lactate concentrations.

## Materials and methods

### Patients

The study received approval of the Ethics Committee (n° 2009-A00105-52) and was conducted in a 15-bed ICU of a teaching hospital (928 beds). Inclusion criteria were ≥18 years of age and ≤80 years of age, and shock. Shock was defined as follows: hypotension requiring fluid infusion and use of vasopressors, and plasma lactate concentrations >2 mmol/L. The shock should be related to sepsis, bleeding, or cardiac dysfunction. The exclusion criteria were pregnancy and patients without a social security number or deprived of freedom. After a next of kin gave informed consent, the patients with shock were prospectively included.

The patients had to be enrolled within six hours after admission to the ICU. All enrolled patients were equipped with a central line and an arterial catheter. Blood samples for *HIF1α *(italic refers to mRNA throughout the manuscript) measurements were collected on PaxgeneTM tubes (BD, Franklin Lakes, NJ, USA) and stored at -80°C until RNA extraction. Sampling was performed at the time of shock detection (H0), and after 1 hour (H1), 3 hours (H3), and 4 hours (H4). These time points were selected to follow the early steps of the interventions in patients with shock.

The following variables were collected: age, sex, body mass index, and admission simplified acute physiology score (SAPS) II [10], sequential organ failure assessment (SOFA) score [11], vital signs, type of shock, type of ventilation, drugs needed for the treatment of shock states, biochemical variables, blood cell count and coagulation variables. In addition, arterial blood gas and plasma lactate concentrations were measured at each time point. Mortality was evaluated at day 28. Data on the duration of mechanical ventilation, vasopressor infusion, and ICU stay were also obtained. Treatment goals were based on most available guidelines [8]. Briefly, mean arterial pressure (MAP) was targeted at ≥65 mmHg, urine flow ≥0.5 ml/kg/hour, and central venous oxygen saturation (ScvO_2_) ≥70%, as described elsewhere [12].

### RNA extraction and quantification of HIF1α variants

Total RNA was isolated using the PAXgene™ Blood RNA Kit (Qiagen, Courtaboeuf, France) according to the manufacturer's instructions. A total of 1µg of RNA was reverse transcribed with 200 UI MMLV Reverse Transcriptase following the EAC (Europe Against Cancer) protocol [13]. The cDNA was diluted in a final volume of 50 µl. Amplification and quantification of *HIF1α *variants were performed as previously described with some modifications of primers and probe sequences (Additional file 1, Figure S1) [14]. Transcripts of the gene coding for TBP (TATA box-binding protein) were also quantified as the endogenous RNA control. Final *HIF1α *mRNA concentrations were expressed in normalized copy numbers as previously described [14].

**Figure 1:**
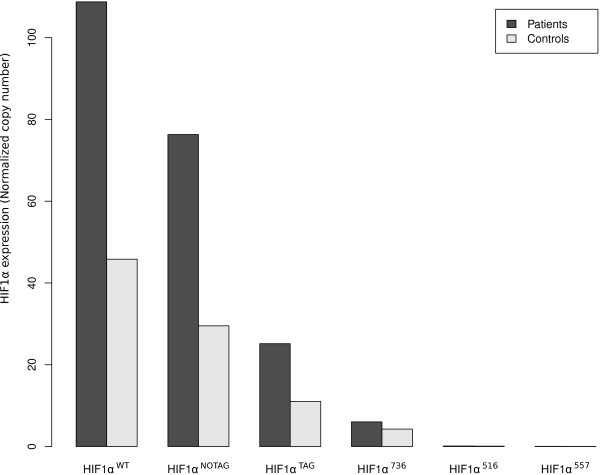
**Expression of different *HIF1α *variants in shock patients (black bars) and controls (grey bars)**.

The gene *HIF1α *is composed of 15 exons, resulting in a principal transcript (*HIF1α*^WT^) [3,15] and seven alternative splice variants which have been reported in human cell lines [16-20]. Amplification of *HIF1α*^WT ^showed that it was expressed by circulating blood cells, as well as the splicing variants *HIF1α*^TAG ^and *HIF1α*^736 ^(Figure 1). *HIF1α*^516 ^and *HIF1α*^557 ^splice variants tested two isoforms coding for negative dominants. These two isoforms were not or were poorly expressed by circulating blood cells. Relative expression of different isoforms was similar between patients and volunteers (Figure 1).

In a subgroup of six patients, at H0, blood was collected from both arterial and venous lines. With regard to the expression of *HIF1a*, no difference was found between the venous and arterial blood samples (data not shown). Then, a group of 11 healthy volunteers, non-smokers, was evaluated for *HIF1α *expression and used as controls.

### Statistical analysis

From previous studies [14,21], 44 patients with shock were required to achieve a predictive value of 90% with a bias <5% and a 5% risk α. Data were analyzed using the software SPSS and R. Quantitative variables are expressed as median and interquartile range. Qualitative variables are expressed as absolute counts and percentages. Differences between groups were tested using non-parametric tests (Mann-Whitney and Kruskall-Wallis tests). A *P *level of 0.05 or less was considered significant.

## Results

### Patient characteristics

Fifty patients with shock (average age 57 (range: 18 to 80 years) and 11 healthy volunteers (average age 50 (range: 29 to 70 years) were prospectively included. Women represented 25% of the cohort of patients and 27% of the cohort of healthy volunteers. The *HIF1α *expression was unaffected by sex (*P *= 0.7) or age (*P *= 0.8). The causes of shock were sepsis, bleeding, and cardiac dysfunction in 39 (78%), 9 (18%), and 2 (4%) cases, respectively (Table 1). Plasma bilirubin concentration and SOFA score differed significantly in survivors and non-survivors (Table 1).

**Table 1:** Characteristics of the patients according to their survival (day 28).

Variables	All patients number = 50	Non-survivors number = 16	Survivors number = 34	*P*
Mechanical ventilation (%)	32 (64)	12 (75)	20 (59)	0.35
Plasma lactate concentrations (mmol/L)	3.1 (1.9-4.8)	3.8 (2.4-7.2)	3 (1.9-4.3)	0.16
MAP (mmHg)	67 (60-72)	70 (52-72(	65 (61-71)	0.89
Plasma creatinine (µmol/L)	112 (85-170)	129 (96-184)	99 (80-155)	0.10
Plasma bilirubin (mmol/L)	11 (6-21)	18 (12-32)	9 (6-18)	0.03
PaO_2_/FiO_2 _ratio	186 (112-374)	169 (97-322)	194 (133-376)	0.44
PaO_2_/FiO_2 _<150	24 (48)	8 (50)	16 (47)	0.96
SAPS 2	46 (32-61)	55 (36-72)	45 (27-55)	0.06
SOFA score	9 (7-11)	10 (9-12)	8 (6-9)	0.003
Hemoglobin level H0 (g/dL)	10.0 (8.7-11.4)	10.6 (9.0-11.3)	10 ([8.5-11.5)	0.48

MAP, mean arterial pressure; PaO_2_/FiO_2, _partial pressure in oxygen related to the inspired fraction of oxygen; SAPS 2, Simplified Acute Physiology Score; SOFA , sequential organ failure assessment.

### HIF1α expression

**Figure 2:**
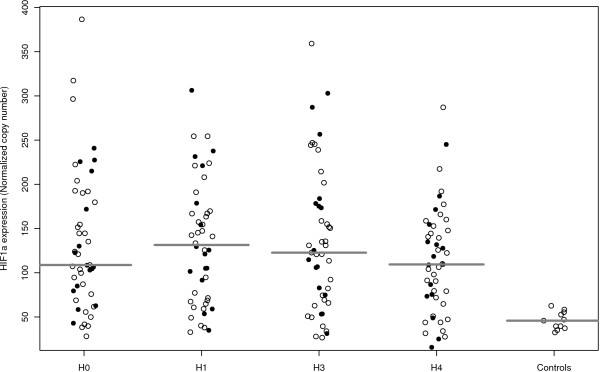
**Expression of *HIF1α *over time in survivors (white circles) and non survivors (black circles)**. The horizontal bar indicates the median for each group.

At any time points of the study period, the expression of *HIF1α *was significantly increased in the patients with shock (Figure 2). At H0, 121 (range: 72 to 168) normalized copies were found in patients with shock, as compared with 46 (range: 38 to 54) normalized copies in healthy volunteers (*P *<0.01). The detailed values for each time point are presented in Table 2. Of note, the expression of *HIF1α *did not differ according to the type of shock (data not shown). We did not find a relation between the expression of *HIF1α *and the absolute number of white blood cells (data not shown).

**Table 2:** Normalized copies of *HIF1α *in patients with shock according to their survival (day 28) and controls.

Time	Patients number = 50	Non-survivors number = 16	Survivors number = 34	*P*
*H0*	109 (78-185)	108 (84-183)	121 (72-185)	0.92
*H1*	132 (69-169)	123 (99-189)	142 (68-167)	0.88
*H3*	123 (72-177)	120 (81-180)	123 (68-157)	0.57
*H4*	109 (73-150)	114 (75-140)	106 (68-150)	0.97

	**Controls** **number = 10**			

*baseline*	46 (38-54)*			

*P < 0.01 as compared with patients at H0.

### HIF1α expression, plasma lactate levels, and outcome

Independently of time points, the *HIF1α *expression did not differ in the survivors and non-survivors (Table 2). In contrast, with the exception of H0, plasma lactate levels were higher in the non-survivors than in the survivors (Table 3). A weak positive correlation was found between *HIF1α *expression and plasma lactate concentrations (r^2 ^= 0.1; *P *= 2.10^-5^).

**Table 3:** Plasma lactate (mmol/L) according to the survival of patients at day 28.

Time	All patients number = 50	Non-survivors number = 16	Survivors number = 34	*P*
*H0*	3.1 (1.9-4.8)	3.6 (2.4-7.2)	3.0 (1.9-4.3)	0.16
*H1*	2.8 (1.8-3.6)	3.4 (2.4-7.3)	2.4 (1.8-3.2)	0.04
*H3*	2.5 (1.7-3.2)	3.4 (2.3-6.3)	2.3 (1.7-2.7)	0.03
*H4*	2.5 (1.9-3.4)	3.7 (2.5-5.3)	2.4 (1.6-3.0)	0.01

No correlation was found between the *HIF1α *expression and admission SAPS 2, shock duration, use of mechanical ventilation, SOFA score, and length of ICU stay. The changes in *HIF1α *expression between H0 and H4 were not predictive of outcome (Figure 2). The *HIF1α *expression was not correlated with hemoglobin, PaO_2_, and PaO_2_/FiO_2 _ratio.

### Expression of HIF1α and response to shock treatment

The expression of *HIF1α *was significantly higher in 24 patients who received more than two liters of fluid expansion: 124 (range: 100 to 168) normalized copies versus 87 (range: 44 to 141) normalized copies (*P *= 0.02). No difference was found according to the type of administered fluid (crystalloid *versus *colloids). The *HIF1α *expression was not correlated with the dose of vasopressors.

## Discussion

The present study is the first to show an increased expression of *HIF1α *in patients with shock, as compared with healthy volunteers. The changes in *HIF1α *expression over time were not correlated with the patient outcome or their treatment responses. Especially, according to our findings, *HIF1α *expression cannot serve to determine the true level of tissue oxygenation.

A significant increase in *HIF1α *expression was observed in the patients who received more than two liters of fluid expansion. Nevertheless, no correlation was found with markers of severity, such as MAP, SAPS2 and SOFA score. This finding invites us to hypothesize that this increase was related to a specific effect of fluid infusion. Among several hypotheses, one may consider that large fluid resuscitation can impair tissue oxygenation [22]. Another explanation would be that fluid administration was related to the severity of vasodilation, which in turn may be related to tissue-hypoxia. Further investigations are needed to clarify this issue. Larger groups of patients should be evaluated in order to elucidate such a specific effect.

HIF1α has an ultra-short half-life [23,24]. One interesting point of the present study is that during the four hours of the study period, the expression of *HIF1α *was stable. Our initial hypothesis was that due to its ultra-short half-life, HIF1α could provide an immediate reflection of tissue oxygenation. We failed to demonstrate this effect or tissue oxygenation remained unaffected by time and treatment steps. A persistent *HIF1α *expression has already been demonstrated in cases of lipopolysaccharide stimulation and during a sustained inflammatory response [25-28]. Such situations are obviously present in the study patients. A major inflammatory response is present in patients with shock due to the ischemia-reperfusion induced by the treatment of shock [23,29-33]. Because plasma lactate concentrations are not only dependent on production but also on its metabolism, we hypothesized that HIF1α would be a better marker. Our study clearly shows that, at the bedside, lactate remains a better marker of shock than *HIF1α*. The quantity of protein may be a more accurate marker than the gene expression. Future studies need to clarify this point.

*HIF1α *is a biomarker of states of cellular hypoxia. Its interest as a marker of outcomes in patients with shock has never been evaluated before. Nevertheless, our results show that, despite attractive speculations about biomarkers, clinical trials are crucial to evaluate their actual role [4,5]. In the present study, the expression of *HIF1α *is markedly increased during shock states. The observed increase could be related to the tissue ischemia of shock states or to the inflammatory response. No relation was found between *HIF1α *expression and oxygenation variables. However, our results show a trend toward an increased expression in patients with low levels of hemoglobin (Hb >8 g/dL: 109 (range: 84 to 174) *HIF1α *copies *versus *Hb <8 g/dL: 161 (range: 74 to 270) *HIF1α *copies; *P *= 0.4). Larger samples of patients would be required in order to validate this trend. The expression of *HIF1α *was wider than expected in our rationale. The wide dispersion of the values may be explained by the ultra-short half-life of HIF1α. This may have affected the power of the study.

In our study, *HIF1α *mRNA expression seems to fail to reflect hypoxia. Several hypotheses may explain this result. First, we measured the expression of HIF1α mRNA in plasma. Actually, in the case of hypoxia, its expression may be more accurate in tissue than in blood. However, regarding our study goals, the collection of tissue biopsy was irrelevant. Second, we may hypothesize that the protein of HIF1α may better reflect tissue hypoxia than its mRNA expression. However, the determination of the protein levels is time consuming, whereas that of RNA levels can be performed in a short time. Our study was aimed at providing an early marker in real-life clinical practice. Finally, divergently from *HIF1α*, plasma lactate levels may reflect pyruvate accumulation rather than cell hypoxia in sepsis and injury [9]. The evaluation of *HIF1α *values beyond four hours may also bring new evidence of its role in patients with shock. Future studies are needed to determine whether its expression during late phases of shock may be related to early interventions. Finally, it is important to consider that the present study focused on blood determinations. This could not reflect with enough accuracy the state of ischemia at the tissue level [21].

## Conclusions

The present study is the first to show the increased expression of *HIF1α*, a transcription factor that controls genes implied in the response to cellular ischemia, in patients with shock. Within the limitations of the study, *HIF1α *expression was not correlated with the outcome of patients. Further studies including larger groups of patients are warranted to clarify this issue.

## Key messages

• Hypoxia-inducible factor 1 alpha (HIF1α) is a transcription factor that controls the expression of genes in response to cellular hypoxia

• *HIF1α *mRNA is elevated in patients with shock, as compared to healthy volunteers

• *HIF1α *expression was not correlated to patient outcome

• *HIF1α *expression over the first hours of shock management was independent of clinical evolution and outcome

• To assess patients with shock, plasma lactate levels seem better than *HIF1α *expression

## Abbreviations

HIF1α: Hypoxia Inducible Factor 1 alpha ; SAPS: Simplified Acute Physiological Score ; SOFA: Sequential Organ Failure Assessment.

## Competing interests

The authors declare that they have no competing interests.

## Authors' contributions

JT, GQ, SW, LZ and VB were involved in the enrollment of patients, the completion of chart report forms, and the collection blood samples. NL and GQ were involved in the enrollment of the healthy donors, the completion of chart report forms, and the collection of blood samples. AB and NB handled the blood samples and performed the molecular analysis. JT, GQ, CM, JG, ML wrote the manuscript. ML, JG, CM designed the study. All authors have read and approved the final manuscript.

## Supplementary Material

Additional file 1 **Figure S1. RT-PCR primers pairs location**. Schematic representation of the location of the various pairs of primers used to amplify several splicing variant of HIF1α. Click here for file
