# Supplementary material for: Hypoxia-inducible factor (HIF1α) gene expression in human shock states
Source: Crit Care. 2012 Jul 10;16(4):R120. doi: 10.1186/cc11414 (PMC3580697; doi:10.1186/cc11414)
Supplement: Additional file 1 — Figure S1. RT-PCR primers pairs location. Schematic representation of the location of the various pairs of primers used to amplify several splicing variant of HIF1α. [file cc11414-S1.PDF]

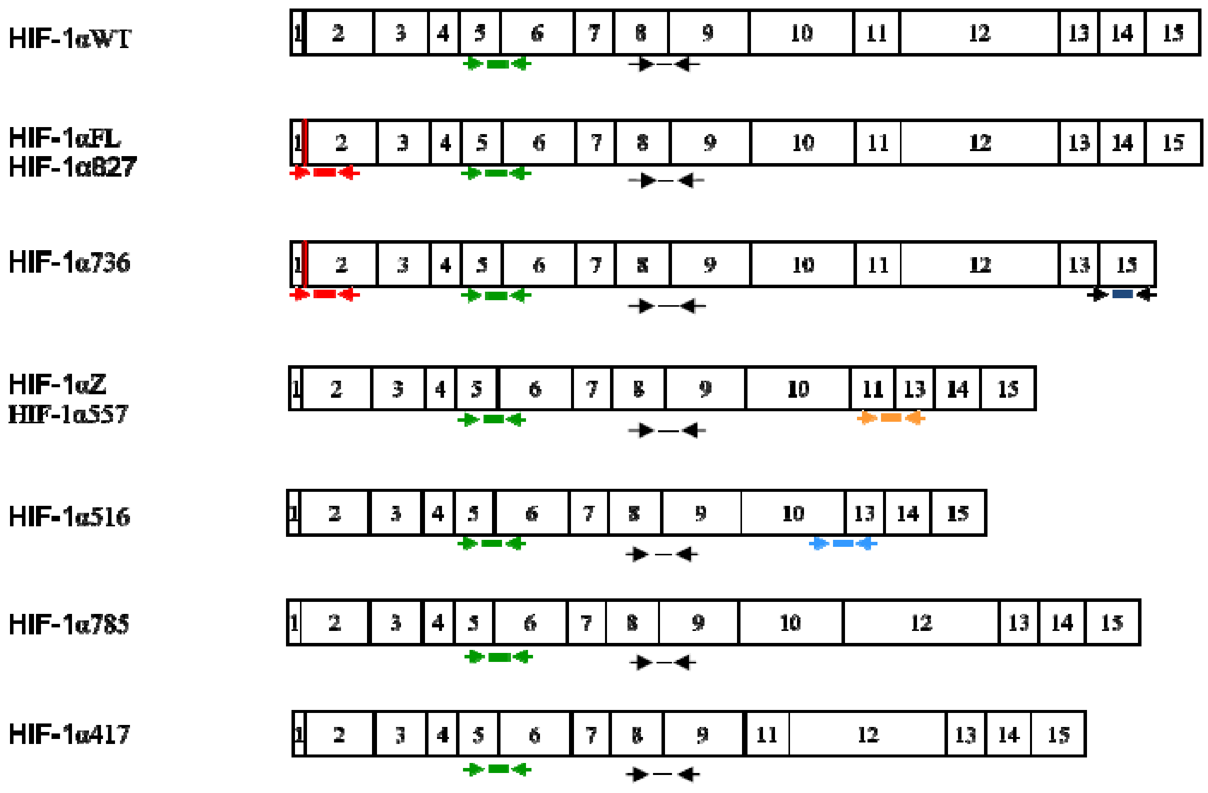

Supplementary figure 1: RT-PCR primers pairs location.

Schematic representation of the location of the various pairs of primers used to amplify several splicing variant of HIF1α
